# Supplementary material for: Next-Generation Mapping of the ACINUS-Mediated Alternative Splicing Machinery and Its Regulation by O-glycosylation in Arabidopsis
Source: bioRxiv. 2025 Sep 19:2025.01.04.631329. Preprint. [Version 3] doi: 10.1101/2025.01.04.631329 (PMC11974692; doi:10.1101/2025.01.04.631329)
Supplement: Supplement 4 [file NIHPP2025.01.04.631329v3-supplement-4.pdf]

bioRxiv preprint doi: https://doi.org/10.1101/2025.01.04.631329; this version posted September 19, 2025. The copyright holder for this preprint (which was not certified by peer review) is the author/funder, who has granted bioRxiv a license to display the preprint in perpetuity. It is made available under aCC-BY 4.0 International license.

Supplementary Figures

Supplementary Fig. S1. **Improved pipeline for retained intron analysis.**

(**A**) Schematic of the improved intron retention analysis pipeline. The left panel illustrates the intron coverage ratio (ICratio) used by Bi et al., 2021, while the right panel introduces the intron depth ratio (IDratio). Key filtering steps are listed, including the application of more stringent thresholds, such as a mean IDratio of at least 15% in at least one group (WT or *acinus pinin*) for statistical analysis.

(**B**) Comparison of parameters used for retained intron analysis with single-end sequencing data from Bi et al., 2021. The updated analysis pipeline applies stricter thresholds, reducing the number of detected altered retained intron events in *acinus pinin* double mutants.

(**C**) Comparison of the detection of retained intron alternations in *acinus pinin* double mutants using new sequencing data and an updated analysis pipeline with different cutoff parameters. A higher IDratio was selected for further analysis, as a lower IDratio may lack biological significance despite statistical significance between WT and the double mutant.

Supplementary Fig. S2: ACINUS-TD, PNN-TD, and SR45-TD fusion proteins localize to the nucleus and complement the leaf shape, rosette size, and flowering phenotypes in corresponding mutants.

bioRxiv preprint doi: https://doi.org/10.1101/2025.01.04.631329; this version posted September 19, 2025. The copyright holder for this preprint (which was not certified by peer review) is the author/funder, who has granted bioRxiv a license to display the preprint in perpetuity. It is made available under aCC-BY 4.0 International license.

(**A**). Spinning disk images of ACINUS-TD, PNN-TD and SR45-TD. Scale =10 µm.
(**B**). Wild type (WT), mutants, and two independent complementation lines of each bait were grown in soil for approximately 4 weeks, showing the complementation of the leaf shape and rosette size phenotypes. Scale = 1cm.
(**C**). The same plants were grown in the greenhouse for about 6 weeks, showing the complementation of the flowering time phenotype by the TurboID fusion proteins. Scale bar=5 cm.

Supplementary Fig. S3. Overlap summary of enriched proteins and representative spectra for identification and quantification of SRPK5 biotinylated peptides.
(**A**) Overlap summary of protein-level enrichment among three baits.
(B) Quantification of SRPK5 biotinylated peptides from reciprocally labeled samples, with MS1 peak matches highlighted. Three peaks corresponding to M, M+1, M+2 peaks are highlighted in red for the upper ^{14}N-labeled peaks and the lower ^{15}N-labeled peaks from forward 1(Fw1) and reverse 1 (Rv1) experiments. Monoisotopic peaks are indicated by arrows, with ^{14}N shown in blue and ^{15}N in red.
(**C-D**). MS2 spectra of *m/z* 586.3122 and 591.9615, 3+ precursors, identified a ^{14}N-labeled (C) or ^{15}N-labeled (D) biotinylated peptide from SRPK5 spanning from amino acid 208 - 221 with a modification at K209, detected in ACINUS-TD enriched samples. The signature ion corresponding to the derivative ion at *m/z* 310.16 (^{14}N) or 311.16 (^{15}N) due to the ammonia loss of the immonium ion of the biotinylated lysine (ImKBio, molecular formula C15 H19 N2 O2 S for ^{14}N-labeled, and C15 H19 N 15N O2 S for ^{15}N-labeled), is labeled with the measured mass.

Supplementary Fig. S4. Cytoscape of the ACINUS-proxiome showing co-transcriptional, co-occurring activity of mRNA processing and high redundancy. The ACINUS-proxiome network is visualized in Cytoscape, integrating TurboID, STRING and GRID data. Nodes represent proteins and edges indicate interactions between them, as shown by gray lines. Proteins from STRING and GRID are circled based on connectivity: nodes with 10-109 edges are bordered in magenta, those with 3-9 degrees are bordered in blue, those with 1-2 edges are bordered in green, and those with no edges are bordered in yellow. Proteins with known or predicted functions are grouped and color-coded as follows: NTC complex filled in gray; A, B/B^{act}, and C complex proteins are filled in green, yellow, and pink, respectively; core and accessory spliceosome components (U1, U2, U4/U5/U6) are filled in darker pink. Proteins involved in transcription and chromatin modeling are filled in light pink, while proteins involved in m6A methylation, 5' capping, 3' polyadenylation and mRNA export are filled in pink, dark green, light green, and light yellow, respectively.

Supplemental Fig. S5. RSB deletion reduces or abolishes interactions and impairs ACINUSΔRSB-TD function in planta.
(A) Quantification of selected proteins over multiple experiments shows reduced or lost interactions in ACINUSΔRSB. Selected examples include several ACINUS interactors and the nuclear standby protein HOP1, which is biotinylated by both baits and controls. The y axis represents the log2-transformed, normalized intensity of each protein across experiments.

bioRxiv preprint doi: https://doi.org/10.1101/2025.01.04.631329; this version posted September 19, 2025. The copyright holder for this preprint (which was not certified by peer review) is the author/funder, who has granted bioRxiv a license to display the preprint in perpetuity. It is made available under aCC-BY 4.0 International license.

(**B**) Transgenic ACINUSΔRSB-TD lines in the *acinus pnn* mutant still show narrow and twisted leaves and overall small rosettes characteristic of double mutants. Four independent lines showing a consistent phenotype.

(**C**) Quantification of leaf length of double mutant and transgenic plants shows that ACINUSΔRSB-TD expression results in larger leaves compared to the *acinus pnn* mutant. y axis shows leaf length.

Supplemental Fig. S6: Common and distinct retained intron events regulated by components in the ACINUS network.

(A) UpSet plot shows the overlapping and distinct retained intron events across all selected mutants.

(**B**) Directionality of regulated retained intron events, comparing *acinus pinin* mutants with other selected mutants.

Supplemental Fig. S7 | The inducible *shSEC RNAi spy-4* line shows seedling lethality.

(**A**) Schematic of Dex-inducible *shSEC RNAi* construct compared to *a2SEC RNAi*. Genomic and cDNA of SEC segments are cloned in a head-to-head orientation to pTA7002 to generate the short hairpin RNAi.

(**B**) Seedling lethality was observed when double mutants were grown directly on Dex-supplemented plates.

(**C**) Progressive phenotype of *shSEC RNAi spy-4* phenotype after Dex treatment, showing reduced root growth by day 3, leaf discoloration by day 5 and complete growth arrest by day 7. (**D**) Quantification of root elongation after transfer on mock and Dex treatment. Root lengths were measured in over 15 seedlings, and statistical analysis was performed using a two-tailed t-test ($p < 0.001$).

Supplementary Fig. S8. PCA analysis of RNA-seq data shows high reproducibility of data sets.

(**A**) PCA clustering shows that the replicates of the WT, *acinus pinin, spy-4,* and *shSEC RNAi* groups clustered closely together, indicating high reproducibility, with minimal effects of DMSO treatment on seedlings.

(**B**) Heatmap showing 8,199 differentially expressed transcripts from Fig. 7D that were altered in the *acinus pinin* mutant or *shSEC RNAi spy-4* with Dex treatment, or both conditions compared to WT. A total of 26,186 transcripts were quantified.

Supplementary Table

Supplementary Table S1: RNA-seq analysis result.

Supplementary Table S2: TurboID-related results. The table includes Table content TurboID summary, evolution conserveness, ACINUS-TD_Protein-level result, PNN-TD_Protein-level result, SR45-TD_Protein-level result, Comparison of ACINUS-TD vs ACINUSΔRSB-TD, ACINUS-TD_Peptide Level, Interpro Results, and Interpro results counts.

Supplementary Table S3: Primers used in this study.

bioRxiv preprint doi: https://doi.org/10.1101/2025.01.04.631329; this version posted September 19, 2025. The copyright holder for this preprint (which was not certified by peer review) is the author/funder, who has granted bioRxiv a license to display the preprint in perpetuity. It is made available under aCC-BY 4.0 International license.

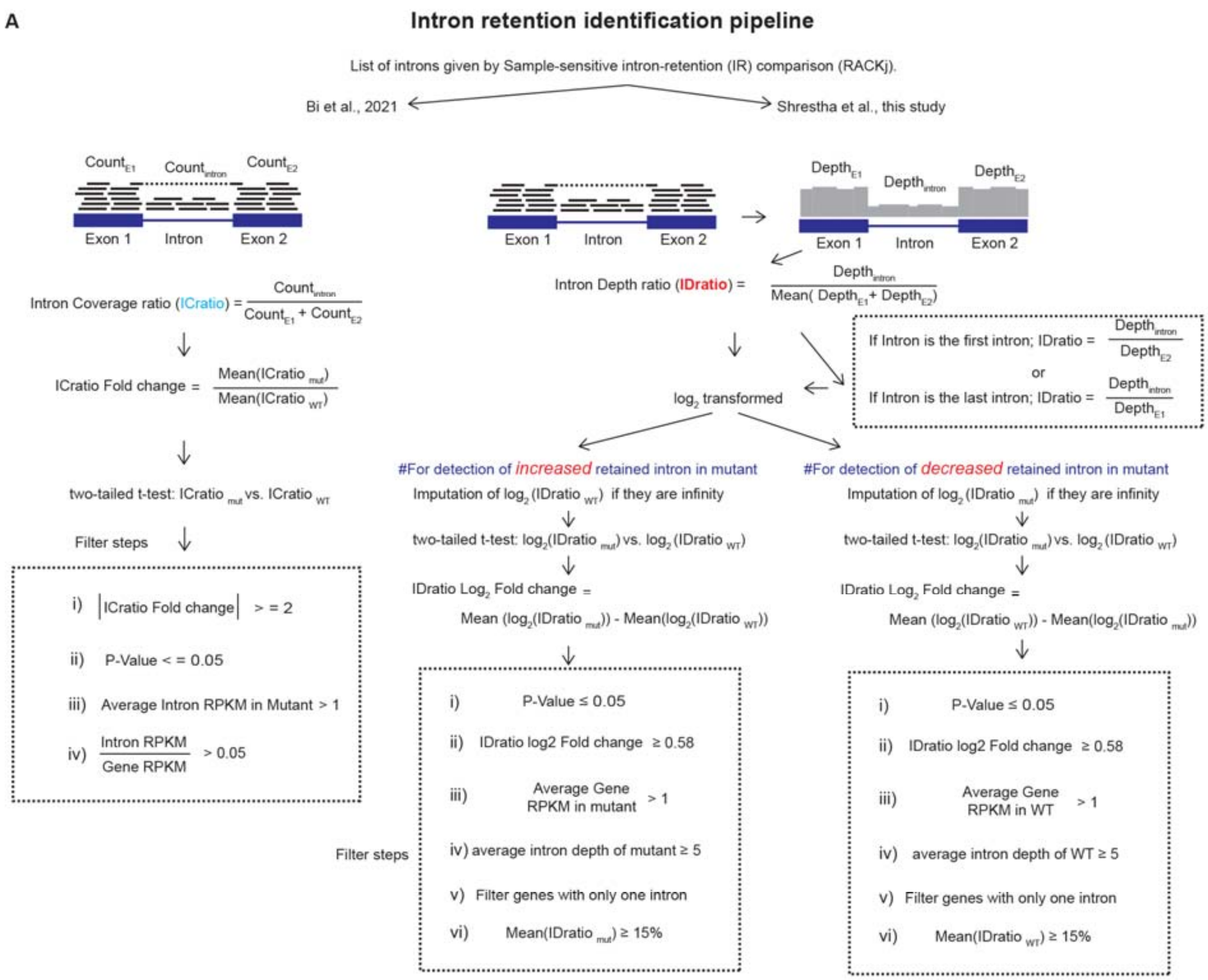

B Data from Bi et al. 2021; single-end sequencing; 100 bp; average coverage of 22.4 million reads. 3 biological replicates each.

Parameters	Increased	Decreased	Total	Method
ICratio ≥ 5%; ≥ 2 fold difference,	258	31	**289**	Old
IDratio ≥ 5%, ≥ 2 fold difference	187	27	**214**	New
IDratio ≥15%, ≥ 2 fold difference	131	19	**150**	New
IDratio ≥15%, ≥ 1.5 fold difference	176	29	**205**	New

C Data from this study, paired-end sequencing; 150bp; average coverage of 57.2 million reads. 4 biological replicates each.

Parameters with new method	Increased	Decreased	Total	Note
IDratio ≥ 5%; ≥ 1.5 fold difference	1,625	574	2,199	
IDratio ≥ 15%; ≥ 1.5 fold difference	**905**	**201**	**1,106**	Chosen for the rest analysis
IDratio ≥ 5%; ≥ 2 fold difference	1,258	350	1,608	
IDratio ≥ 15%; ≥ 2 fold difference	668	113	781	

Supplementary Fig. S1. **Improved pipeline for retained intron analysis.**
(**A**) Schematic of the improved intron retention analysis pipeline. The left panel illustrates the intron coverage ratio (ICratio) used by Bi et al., 2021, while the right panel introduces the intron depth ratio (IDratio). Key filtering steps are listed, including the application of more stringent thresholds, such as a mean IDratio of at least 15% in at least one group (WT or *acinus pinin*) for statistical analysis.

bioRxiv preprint doi: https://doi.org/10.1101/2025.01.04.631329; this version posted September 19, 2025. The copyright holder for this preprint (which was not certified by peer review) is the author/funder, who has granted bioRxiv a license to display the preprint in perpetuity. It is made available under aCC-BY 4.0 International license.

(**B**) Comparison of parameters used for retained intron analysis with single-end sequencing data from Bi et al., 2021. The updated analysis pipeline applies stricter thresholds, reducing the number of detected altered retained intron events in *acinus pinin* double mutants.
(**C**) Comparison of the detection of retained intron alternations in *acinus pinin* double mutants using new sequencing data and an updated analysis pipeline with different cutoff parameters. A higher IDratio was selected for further analysis, as a lower IDratio may lack biological significance despite statistical significance between WT and the double mutant.

bioRxiv preprint doi: https://doi.org/10.1101/2025.01.04.631329; this version posted September 19, 2025. The copyright holder for this preprint (which was not certified by peer review) is the author/funder, who has granted bioRxiv a license to display the preprint in perpetuity. It is made available under aCC-BY 4.0 International license.

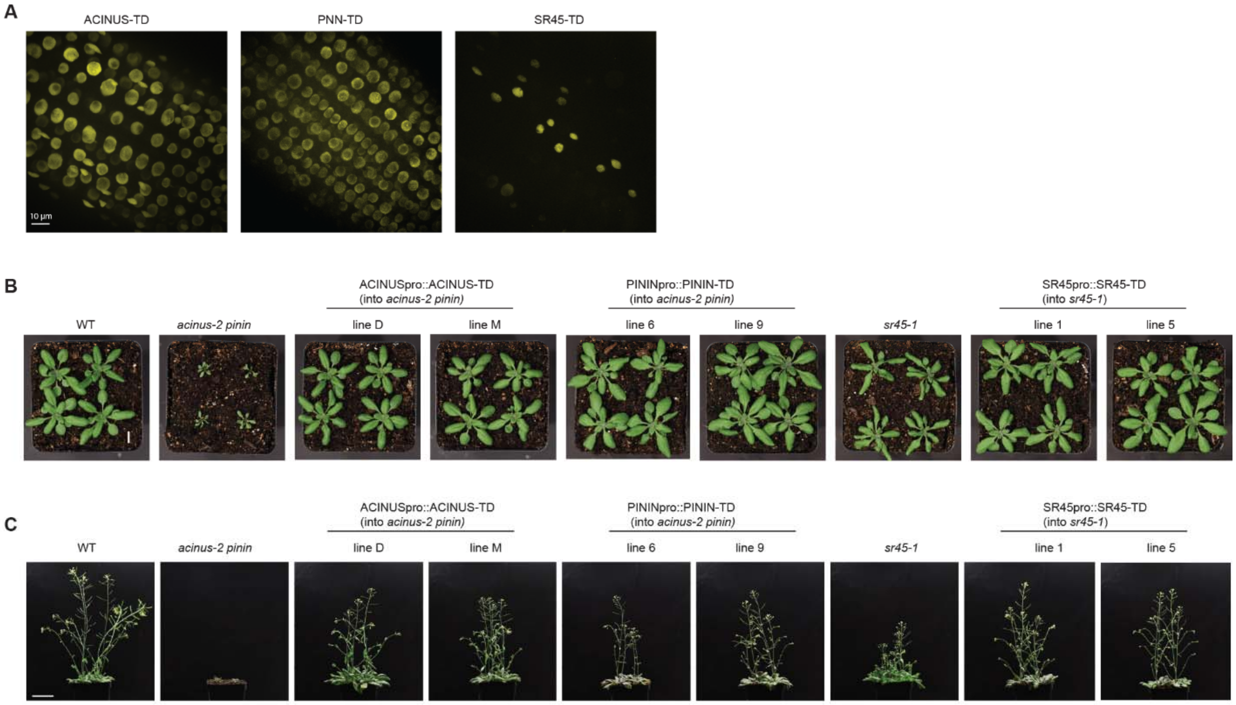

Supplementary Fig. S2: ACINUS-TD, PNN-TD, and SR45-TD fusion proteins localize to the nucleus and complement the leaf shape, rosette size, and flowering phenotypes in corresponding mutants.

(**A**). Spinning disk images of ACINUS-TD, PNN-TD and SR45-TD. Scale =10 µm.

(**B**). Wild type (WT), mutants, and two independent complementation lines of each bait were grown in soil for approximately 4 weeks, showing the complementation of the leaf shape and rosette size phenotypes. Scale = 1cm.

(**C**). The same plants were grown in the greenhouse for about 6 weeks, showing the complementation of the flowering time phenotype by the TurboID fusion proteins. Scale bar=5 cm.

bioRxiv preprint doi: https://doi.org/10.1101/2025.01.04.631329; this version posted September 19, 2025. The copyright holder for this preprint (which was not certified by peer review) is the author/funder, who has granted bioRxiv a license to display the preprint in perpetuity. It is made available under aCC-BY 4.0 International license.

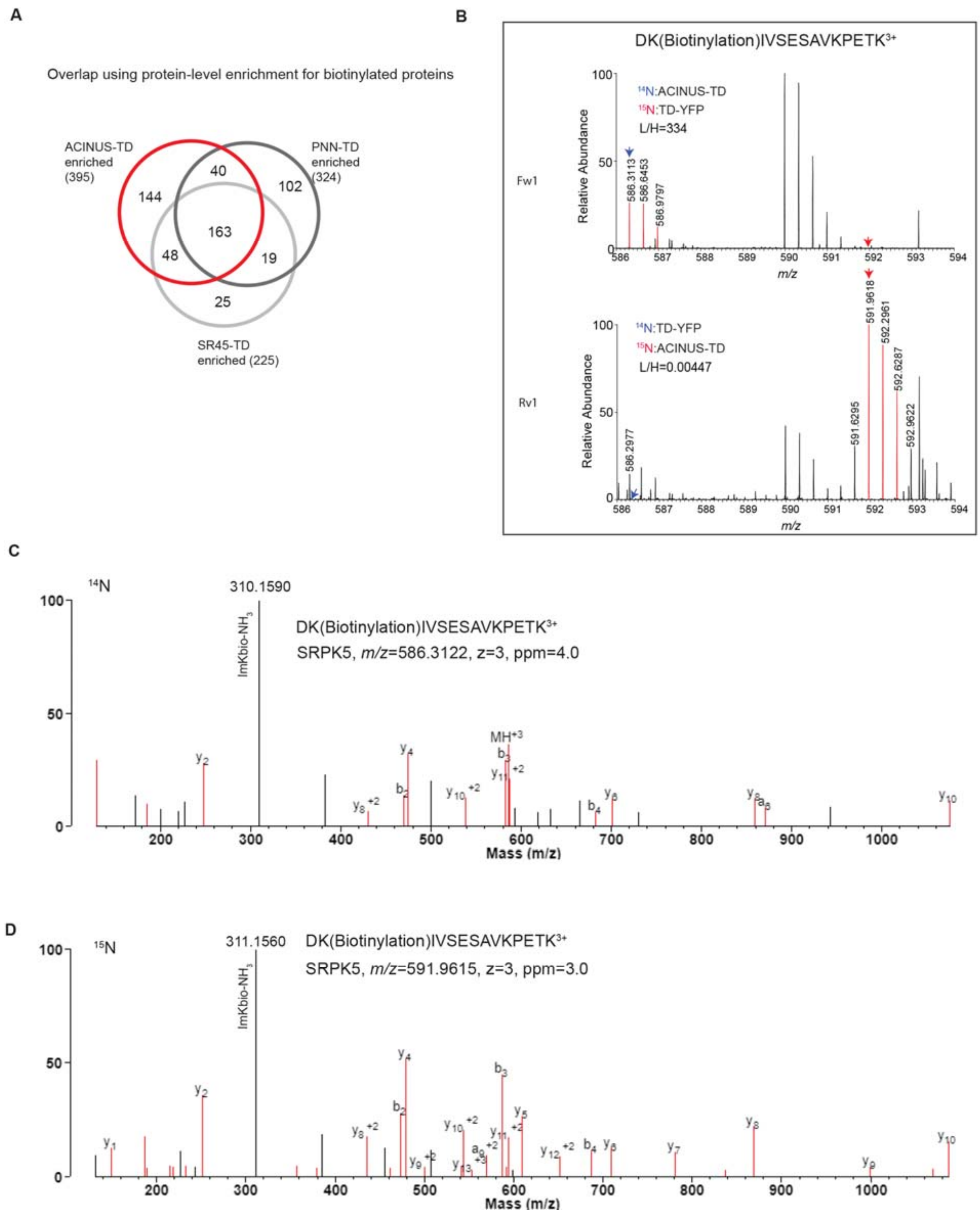

Supplementary Fig. S3. Overlap summary of enriched proteins and representative spectra for identification and quantification of SRPK5 biotinylated peptides.

(**A**) Overlap summary of protein-level enrichment among three baits.

(B) Quantification of SRPK5 biotinylated peptides from reciprocally labeled samples, with MS1 peak matches highlighted. Three peaks corresponding to M, M+1, M+2 peaks are highlighted in red for the

bioRxiv preprint doi: https://doi.org/10.1101/2025.01.04.631329; this version posted September 19, 2025. The copyright holder for this preprint (which was not certified by peer review) is the author/funder, who has granted bioRxiv a license to display the preprint in perpetuity. It is made available under aCC-BY 4.0 International license.

upper ^{14}N-labeled peaks and the lower ^{15}N-labeled peaks from forward 1(Fw1) and reverse 1 (Rv1) experiments. Monoisotopic peaks are indicated by arrows, with ^{14}N shown in blue and ^{15}N in red. (**C**-**D**). MS2 spectra of *m/z* 586.3122 and 591.9615, 3+ precursors, identified a ^{14}N-labeled (C) or ^{15}N-labeled (D) biotinylated peptide from SRPK5 spanning from amino acid 208 - 221 with a modification at K209, detected in ACINUS-TD enriched samples. The signature ion corresponding to the derivative ion at *m/z* 310.16 (^{14}N) or 311.16 (^{15}N) due to the ammonia loss of the immonium ion of the biotinylated lysine (ImKBio, molecular formula C15 H19 N2 O2 S for ^{14}N-labeled, and C15 H19 N 15N O2 S for ^{15}N-labeled), is labeled with the measured mass.

bioRxiv preprint doi: https://doi.org/10.1101/2025.01.04.631329; this version posted September 19, 2025. The copyright holder for this preprint (which was not certified by peer review) is the author/funder, who has granted bioRxiv a license to display the preprint in perpetuity. It is made available under aCC-BY 4.0 International license.

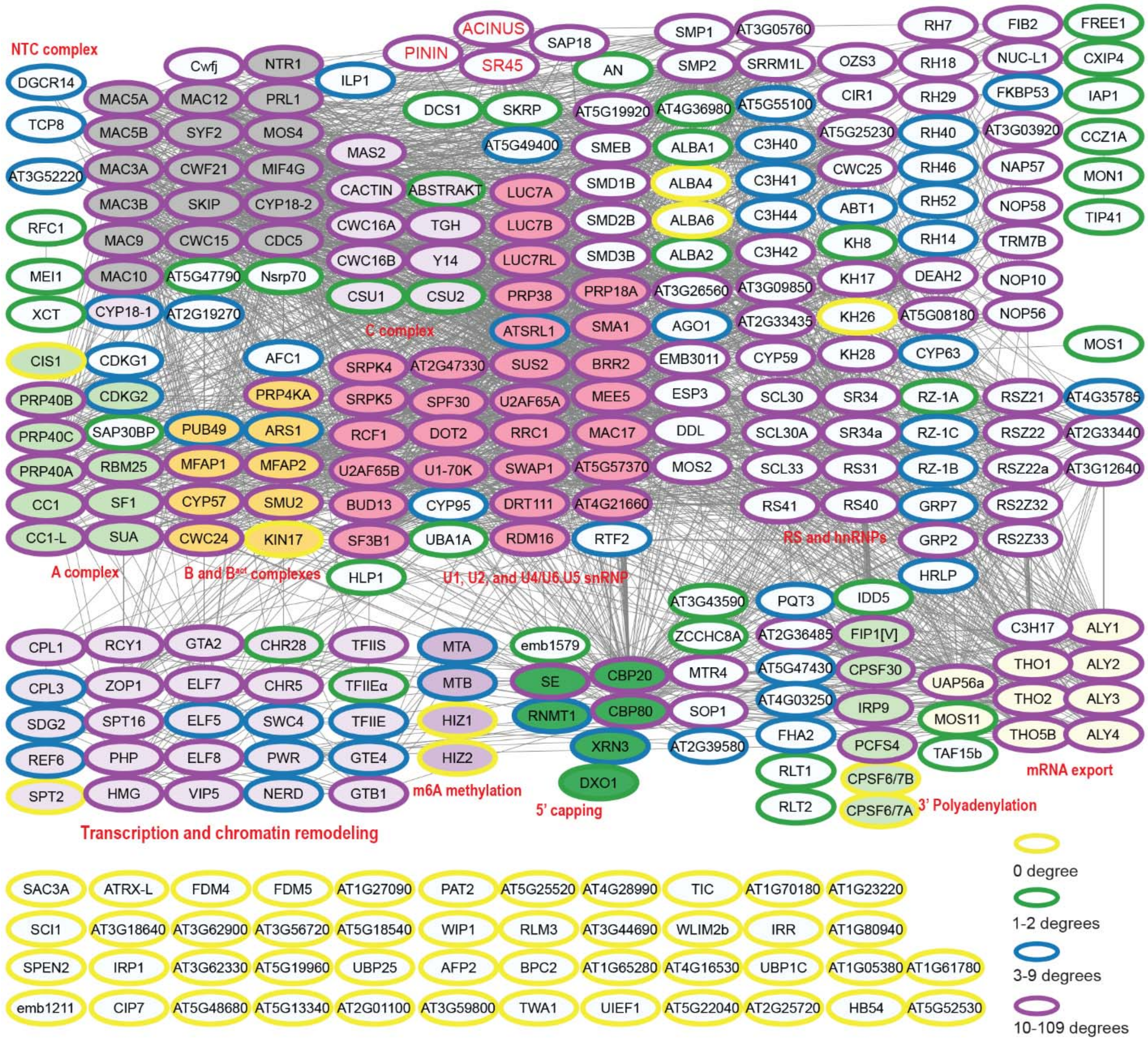

Supplementary Fig. S4. Cytoscape of the ACINUS-proxiome showing co-transcriptional, co-occurring activity of mRNA processing and high redundancy. The ACINUS-proxiome network is visualized in Cytoscape, integrating TurboID, STRING and GRID data. Nodes represent proteins and edges indicate interactions between them, as shown by gray lines. Proteins from STRING and GRID are circled based on connectivity: nodes with 10-109 edges are bordered in magenta, those with 3-9 degrees are bordered in blue, those with 1-2 edges are bordered in green, and those with no edges are bordered in yellow. Proteins with known or predicted functions are grouped and color-coded as follows: NTC complex filled in gray; A, B/B^{act}, and C complex proteins are filled in green, yellow, and pink, respectively; core and accessory spliceosome components (U1, U2, U4/U5/U6) are filled in darker pink. Proteins involved in transcription and chromatin modeling are filled in light pink, while proteins involved in m6A methylation, 5' capping, 3' polyadenylation and mRNA export are filled in pink, dark green, light green, and light yellow, respectively.

bioRxiv preprint doi: https://doi.org/10.1101/2025.01.04.631329; this version posted September 19, 2025. The copyright holder for this preprint (which was not certified by peer review) is the author/funder, who has granted bioRxiv a license to display the preprint in perpetuity. It is made available under aCC-BY 4.0 International license.

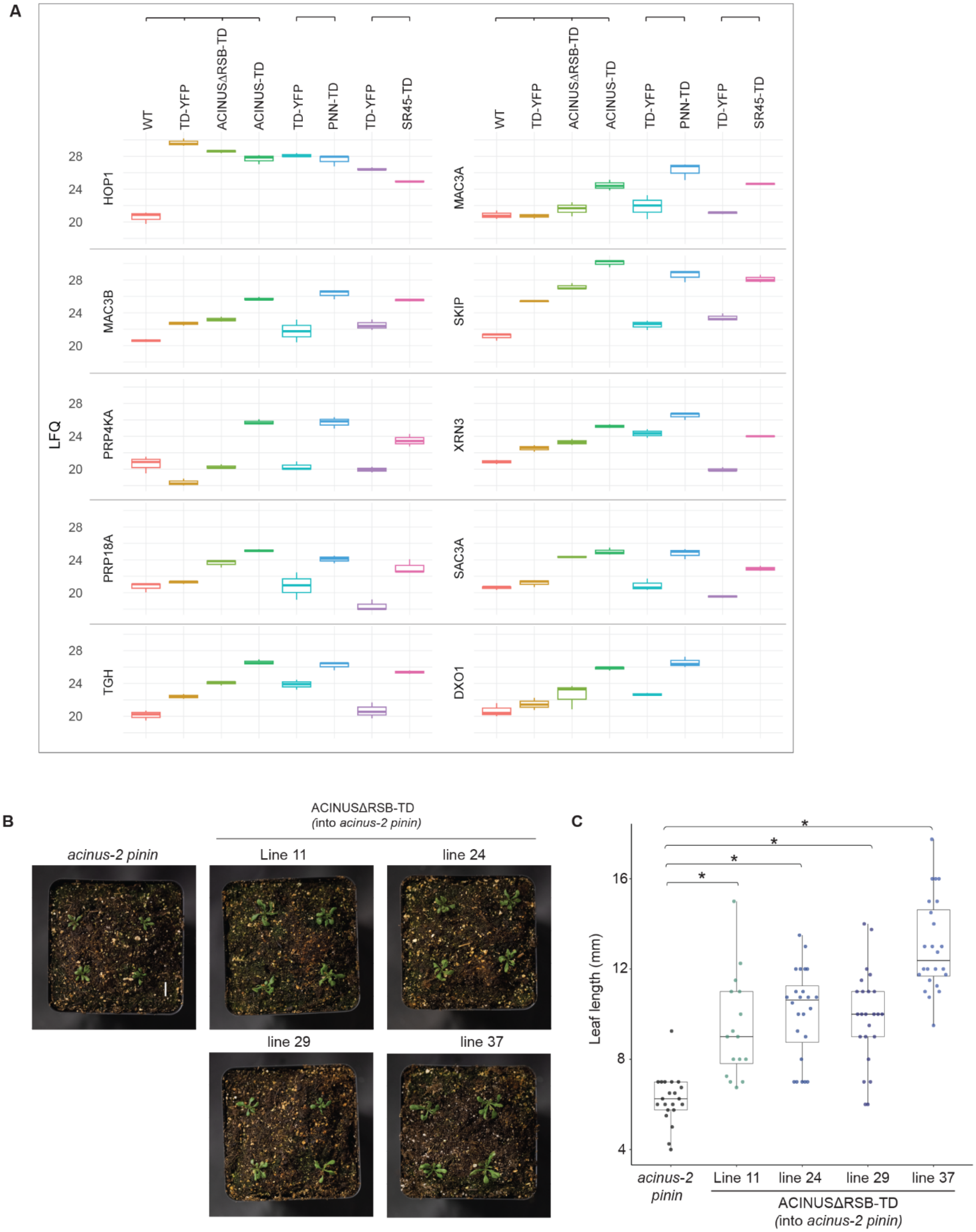

Supplemental Fig. S5. RSB deletion reduces or abolishes interactions and impairs ACINUSΔRSB-TD function in planta.

bioRxiv preprint doi: https://doi.org/10.1101/2025.01.04.631329; this version posted September 19, 2025. The copyright holder for this preprint (which was not certified by peer review) is the author/funder, who has granted bioRxiv a license to display the preprint in perpetuity. It is made available under aCC-BY 4.0 International license.

(A) Quantification of selected proteins over multiple experiments shows reduced or lost interactions in ACINUSΔRSB. Selected examples include several ACINUS interactors and the nuclear standby protein HOP1, which is biotinylated by both baits and controls. The y axis represents the log2-transformed, normalized intensity of each protein across experiments.
(**B**) Transgenic ACINUSΔRSB-TD lines in the *acinus pnn* mutant still show narrow and twisted leaves and overall small rosettes characteristic of double mutants. Four independent lines showing a consistent phenotype.
(**C**) Quantification of leaf length of double mutant and transgenic plants shows that ACINUSΔRSB-TD expression results in larger leaves compared to the *acinus pnn* mutant. y axis shows leaf length.

bioRxiv preprint doi: https://doi.org/10.1101/2025.01.04.631329; this version posted September 19, 2025. The copyright holder for this preprint (which was not certified by peer review) is the author/funder, who has granted bioRxiv a license to display the preprint in perpetuity. It is made available under aCC-BY 4.0 International license.

A

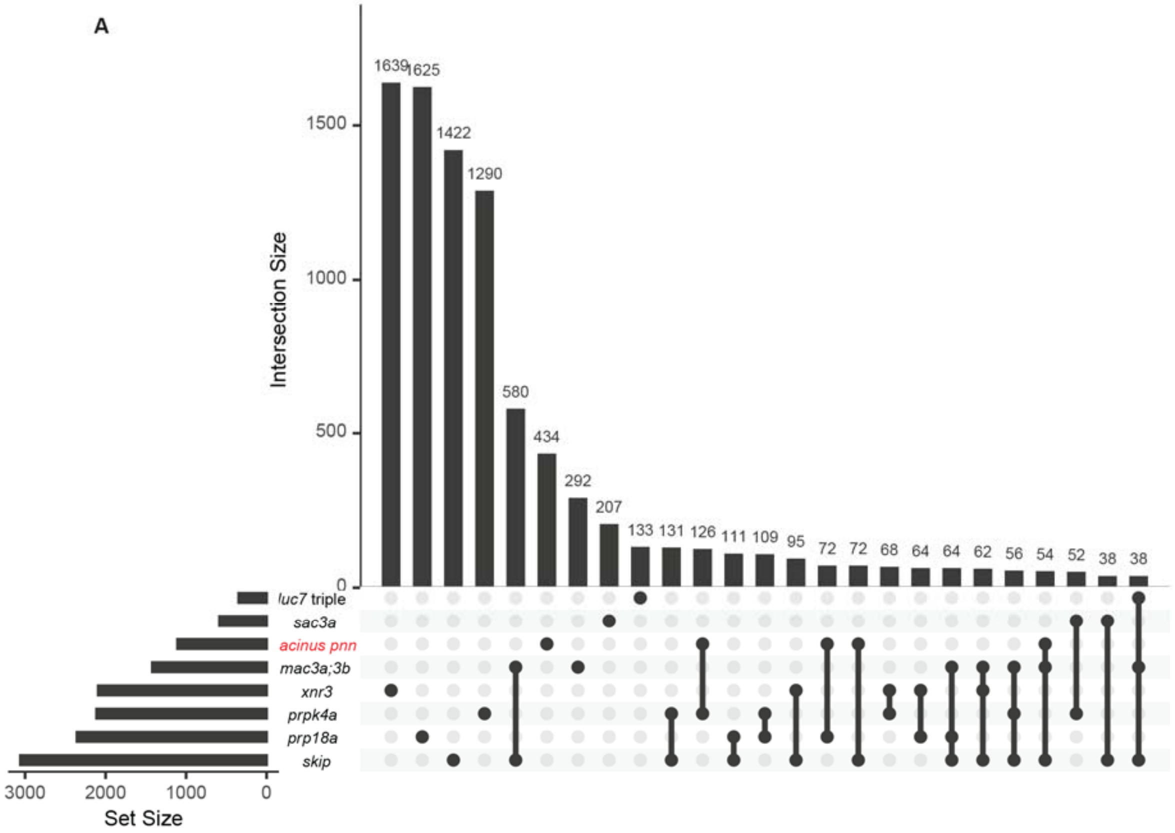

B

		up in *ap* (905)	down in *ap* (201)
skip			
↑	(3025)	252	29
↓	(33)	0	7
mac3a mac3b			
↑	(1391)	137	14
↓	(28)	1	3
prp18a			
↑	(2087)	115	16
↓	(269)	39	20
prp4ka			
↑	(2055)	217	29
↓	(57)	8	5
luc7 triple			
↑	(225)	32	9
↓	(121)	5	11
xrn3			
↑	(1692)	39	4
↓	(399)	6	3
sac3a			
↑	(447)	89	10
↓	(137)	5	31

bioRxiv preprint doi: https://doi.org/10.1101/2025.01.04.631329; this version posted September 19, 2025. The copyright holder for this preprint (which was not certified by peer review) is the author/funder, who has granted bioRxiv a license to display the preprint in perpetuity. It is made available under aCC-BY 4.0 International license.

Supplemental Fig. S6: Common and distinct retained intron events regulated by components in the ACINUS network.

(A) UpSet plot shows the overlapping and distinct retained intron events across all selected mutants.

(**B**) Directionality of regulated retained intron events, comparing *acinus pinin* mutants with other selected mutants.

bioRxiv preprint doi: https://doi.org/10.1101/2025.01.04.631329; this version posted September 19, 2025. The copyright holder for this preprint (which was not certified by peer review) is the author/funder, who has granted bioRxiv a license to display the preprint in perpetuity. It is made available under aCC-BY 4.0 International license.

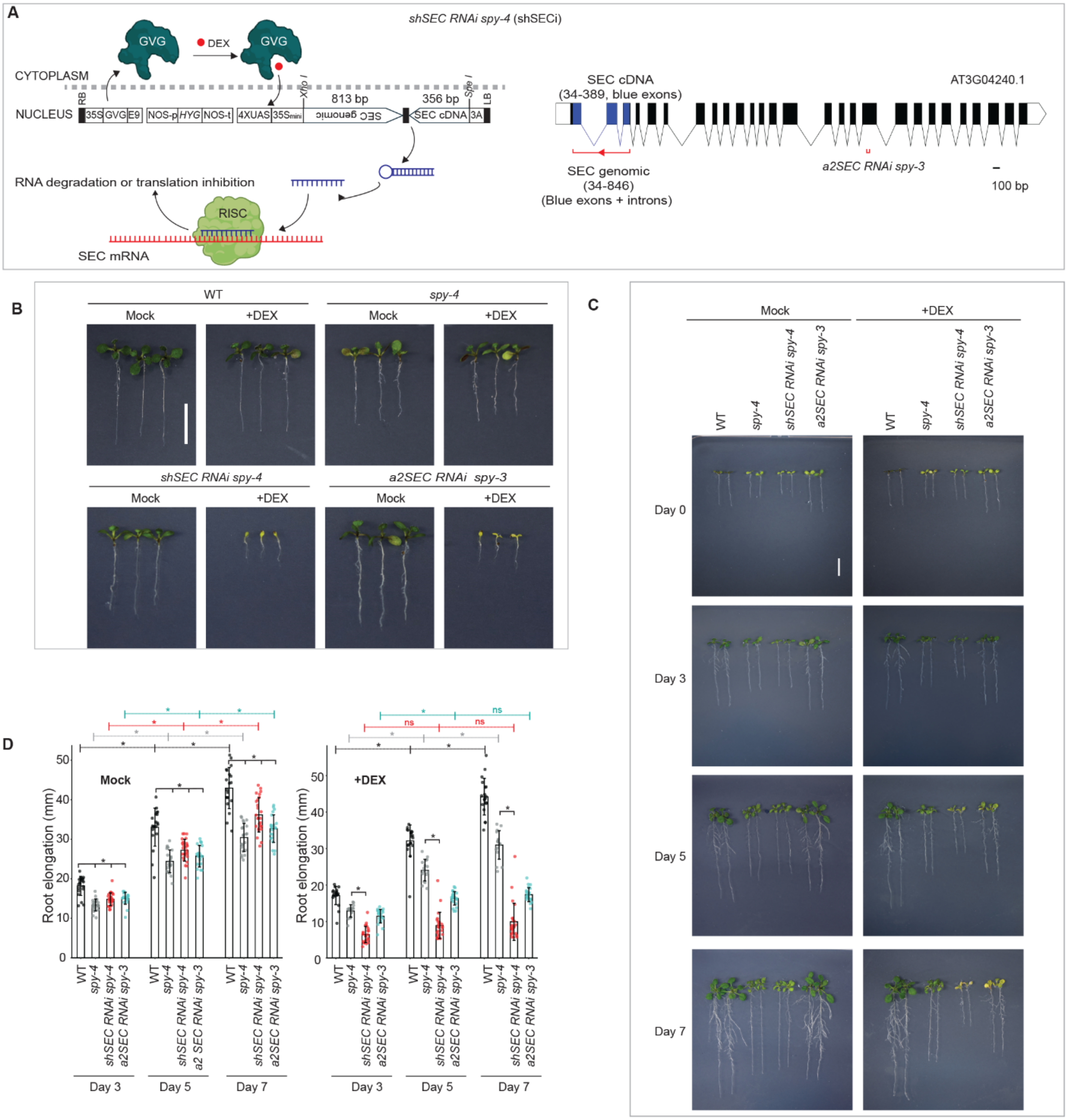

Supplemental Fig. S7 | The inducible *shSEC RNAi spy-4* line shows seedling lethality.
(**A**) Schematic of Dex-inducible *shSEC RNAi* construct compared to *a2SEC RNAi*. Genomic and cDNA of SEC segments are cloned in a head-to-head orientation to pTA7002 to generate the short hairpin RNAi.
(**B**) Seedling lethality was observed when double mutants were grown directly on Dex-supplemented plates.
(**C**) Progressive phenotype of *shSEC RNAi spy-4* phenotype after Dex treatment, showing reduced root growth by day 3, leaf discoloration by day 5 and complete growth arrest by day 7. (**D**) Quantification of root elongation after transfer on mock and Dex treatment. Root lengths were measured in over 15 seedlings, and statistical analysis was performed using a two-tailed t-test ($p < 0.001$).

bioRxiv preprint doi: https://doi.org/10.1101/2025.01.04.631329; this version posted September 19, 2025. The copyright holder for this preprint (which was not certified by peer review) is the author/funder, who has granted bioRxiv a license to display the preprint in perpetuity. It is made available under aCC-BY 4.0 International license.

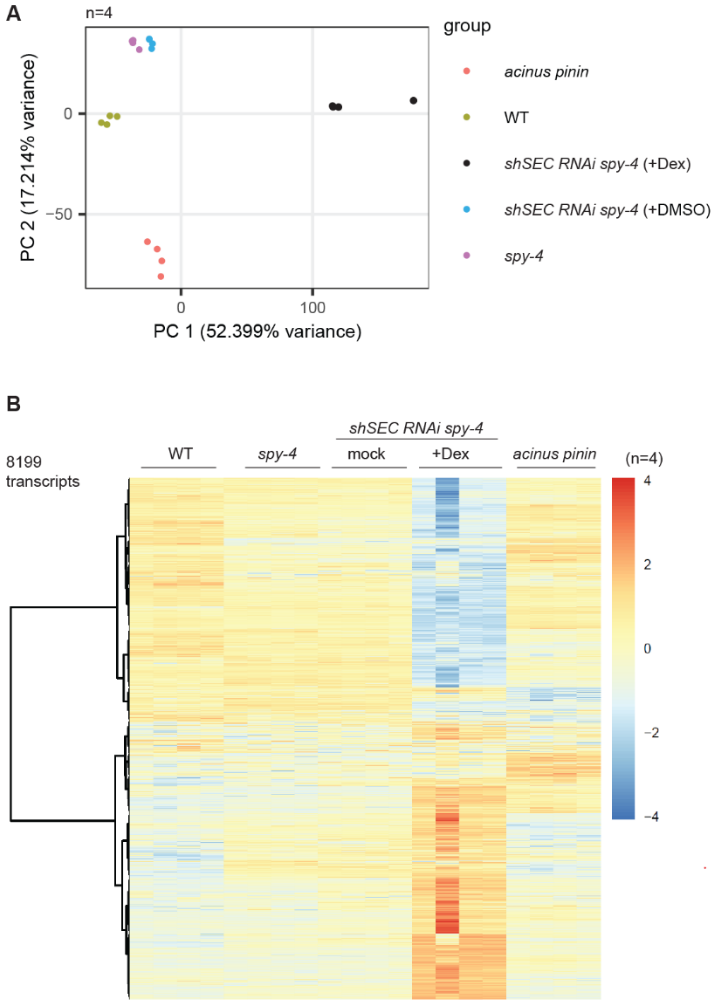

Supplementary Fig. S8. PCA analysis of RNA-seq data shows high reproducibility of data sets.
(**A**) PCA clustering shows that the replicates of the WT, *acinus pinin*, *spy-4*, and *shSEC RNAi* groups clustered closely together, indicating high reproducibility, with minimal effects of DMSO treatment on seedlings.
(**B**) Heatmap showing 8,199 differentially expressed transcripts from Fig. 7D that were altered in the *acinus pinin* mutant or *shSEC RNAi spy-4* with Dex treatment, or both conditions compared to WT. A total of 26,186 transcripts were quantified.
